# Developmental modeling of hepatogenesis using obese iPSCs-hepatocyte differentiation uncovers pathological features

**DOI:** 10.1038/s41419-022-05125-9

**Published:** 2022-08-01

**Authors:** Divya Saro Varghese, Thilina T. Alawathugoda, Muhammad Abid Sheikh, Anil Kumar Challagandla, Bright Starling Emerald, Suraiya A. Ansari

**Affiliations:** 1 Department of Biochemistry and Molecular Biology, College of Medicine and Health Sciences, United Arab Emirates University, P.O. Box 17666, Al Ain, United Arab Emirates; 2 Department of Anatomy, College of Medicine and Health Sciences, United Arab Emirates University, P.O. Box 17666, Al Ain, United Arab Emirates; 3 Zayed Center for Health Sciences, United Arab Emirates University, Al Ain, Abu Dhabi UAE

**Keywords:** Differentiation, Developmental biology

## Abstract

Obesity is a multigene disorder. However, in addition to genetic factors, environmental determinants also participate in developing obesity and related pathologies. Thus, obesity could be best described as a combination of genetic and environmental perturbations often having its origin during the early developmental period. Environmental factors such as energy-dense food and sedentary lifestyle are known to be associated with obesogenicity. However, the combinatorial effects of gene-environment interactions are not well understood. Understanding the role of multiple genetic variations leading to subtle gene expression changes is not practically possible in monogenic or high-fat-fed animal models of obesity. In contrast, human induced pluripotent stem cells (hiPSCs) from individuals with familial obesity or an obesogenic genotype could serve as a good model system. Herein, we have used hiPSCs generated from normal and genetically obese subjects and differentiated them into hepatocytes in cell culture. We show that hepatocytes from obese iPSCs store more lipids and show increased cell death than normal iPSCs. Whole transcriptome analyses in both normal and obese iPSCs treated with palmitate compared to control revealed LXR-RXR and hepatic fibrosis pathways were enriched among other pathways in obese iPSCs compared to normal iPSCs. Among other genes, increased CD36 and CAV1 expression and decreased expression of CES1 in obese iPSCs could have been responsible for excess lipid accumulation, resulting in differential expression of genes associated with hepatic fibrosis, a key feature of non-alcoholic fatty liver disease (NAFLD). Our results demonstrate that iPSCs derived from genetically obese subjects could serve as an excellent model to understand the effects of this multigene disorder on organ development and may uncover pathologies of NAFLD, which is highly associated with obesity.

## Introduction

Obesity is a multigene disorder where subtle changes in the expression of several genes with roles in cellular metabolism and inflammation lead to obesity and metabolic syndrome. These alterations in gene expression could happen due to genetic variations and adverse environmental conditions such as high-fat diet feeding and sedentary lifestyle. The development of obesity, however, may combine genetic variations with other adverse metabolic conditions, making certain individuals more susceptible to the development of obesity than others. Thus, the pathology of obesity, especially in childhood or early adulthood, may have a developmental origin. Obesity is intimately linked to chronic liver diseases, including insulin resistance and non-alcoholic fatty liver disease (NAFLD). It is important to note that the prevalence of NAFLD in children is increasingly being recognized, which could progress to liver fibrosis and cirrhosis in early adulthood [1]. Therefore, early detection and possible intervention could play a key role in managing and preventing end-stage liver disease in adulthood. However, the unavailability of sufficient human fetal liver tissues or tissues from early childhood is a major limitation of understanding human-specific pathological mechanisms and novel therapeutic developments.

Induced pluripotent stem cells (iPSCs) could serve as an excellent alternative to human tissues and are being utilized extensively to model disease and development in a cell culture dish [2, 3]. Derivation of iPSCs from normal and genetically susceptible obese subjects has been recently reported [4]. hiPSCs generated from super-obese (BMI ≥ 50) and normal (BMI ≤ 25) subjects were differentiated into hypothalamic-like neuronal cells to investigate the mechanisms of metabolic dysregulation impacting the CNS [4]. However, such iPSCs have not been used so far to study the effect of obesogenic genotype on hepatocyte development, differentiation and pathology.

Herein, we have used hiPSCs from normal and obese subjects generated by Rajamani et al. [4] and differentiated them into hepatocytes using our established hepatocyte differentiation protocol [5, 6]. NAFLD is a heritable disease [7] however, the role of unhealthy lifestyles such as high-fat diet and sedentary life are contributory factors to the development of metabolic syndrome and NAFLD [8, 9]. Therefore, in this study, our goal was to understand the effect of an obesogenic genotype combined with dyslipidemia (high free fatty acid levels) on hepatocyte differentiation and function. These hiPSCs were characterized at four key stages of hepatocyte differentiation by lineage-specific marker expression analysis and hepatocyte-specific functional assays. We found increased lipid accumulation and cell death in obese iPSCs compared to normal on the last two stages of hepatocyte differentiation. Furthermore, whole transcriptome analysis from these four stages of hepatocyte differentiation revealed differential expression of genes from LXR/RXR, LXR/FXR as well as hepatic fibrosis/hepatic stellate cell activation pathways in obese iPSCs and in both normal and obese iPSCs when treated with palmitate, suggesting a molecular signature pathological in nature.

## Results

### Phenotypic characterization of obese human iPSCs-derived hepatocytes differentiated in vitro

**Fig. 1:**
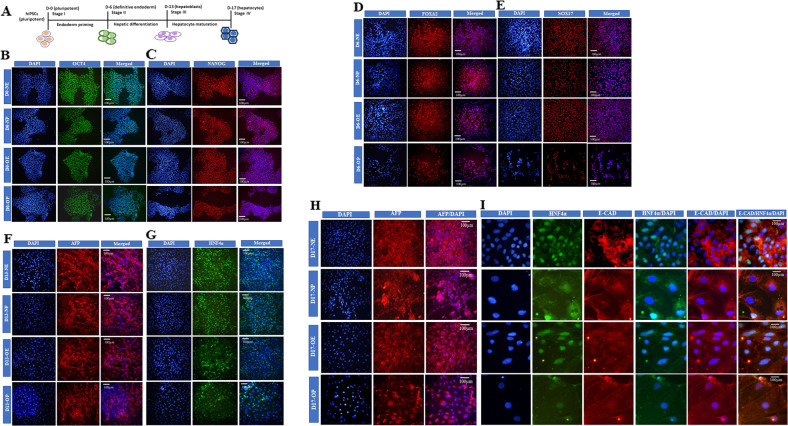
In vitro hepatic differentiation of normal and obese iPSCs in control and treated with palmitate. **A** Schematic representation of stage-specific differentiation of normal and obese iPSCs into hepatocytes. Immunostaining images showing the expression of stage-specific markers in ethanol and palmitate treated control and obese iPSCs-derived cells at various stages of differentiation. Pluripotency markers **B** OCT4 and **C** NANOG expression on day 0 of differentiation. Definitive endoderm markers **D** FOXA2 and **E** SOX17 were expressed on day 6 of differentiation. Hepatoblast markers **F** AFP-1 and **G** HNF4α expression on day 13 of differentiation. Expression of **H** AFP-1 **I** HNAF4α and E-CAD (E-Cadherin) in hepatocytes on day 17 of differentiation. Images were captured using the ×40 objective of Olympus IX70 Inverted Fluorescence Microscope. Scale bar = 100 µM. NE Normal iPSC-derived, ethanol-treated, NP- Normal iPSC-derived, palmitate treated, OE Obese iPSC-derived, ethanol-treated; OP Obese iPSC-derived, palmitate treated.

We used a four-step hepatocyte differentiation method (1. pluripotent, 2. definitive endoderm, 3. hepatoblasts, and 4. mature hepatocyte) which recapitulates the early stages of embryonic hepatogenesis. This protocol is based on previously published protocols but with several modifications [5] to achieve higher levels of expression of lineage-specific markers suitable for hepatocyte development and disease modeling. We performed an in vitro hepatocyte differentiation of hiPSCs from normal and super-obese individuals [4] and analyzed phenotypic changes during differentiation in the absence or presence of 100 μM saturated fatty acid, palmitate to generate a metabolically altered environment. Solvent (ethanol) treated cells were used as control. The expression of stage-specific markers during the entire course of differentiation was tracked and compared in all four stages of hepatocyte differentiation. Neither the administration of excess saturated fatty acids nor the genotype of the iPSCs altered the expression of the pluripotency markers OCT4 and NANOG in undifferentiated cells (day 0, stage-1) (Fig. 1A, B). On day 6 (stage-2) of differentiation, all cells stained positive for endoderm-specific markers, FOXA2 and SOX17 suggestive of definitive endoderm (DE) fate. The cytotoxic effect of palmitic acid treatment was evident in obese iPSCs’ derived DE cells as fewer cells survived the treatment (Fig. 1C, D). In the hepatoblasts (stage-3, day 13), the AFP expression remained almost unaltered in all four conditions, but fewer cells expressed HNF4α in treated and nontreated hepatoblasts derived from genetically obese iPSCs. Thus, although palmitic acid did not affect the expression patterns of HNF4α in normal hepatoblasts, it was reduced in treated obese hepatoblasts compared to all other three conditions, i.e., normal (control and palmitate treated) and obese (palmitate treated) cells (Fig. 1E, F). On day 17 (stage-4) of differentiation, AFP expression was acutely impaired in palmitate-treated normal and obese hepatocytes, with more severe effectivity in obese iPSCs’ derived hepatocytes (Fig. 1G). The nuclear factor HNF4α localized to the nuclei, and the E-Cadherin (E-Cad) was expressed along cell membranes, clearly demarcating the polygonal morphology of the hepatocytes in normal nontreated cells. While the expression of HNF4α was predominantly nuclear in nontreated obese hepatocytes, abnormal localization of HNF4α was also observed in the cytoplasm and the membrane in the palmitate treated normal and obese hepatocytes. Except for the nontreated normal iPSCs-derived hepatocytes, the expression of E-Cad was greatly reduced in the remaining three conditions, despite the polygonal morphology being exhibited in all these mature hepatocytes (Fig. 1H).

### Global transcriptome analysis in normal and obese iPSCs

We have performed high-throughput RNA-seq from four stages of hepatocyte differentiation as explained above to obtain temporal gene expression profile during iPSCs’ differentiation into hepatocytes to identify genes and non-coding RNAs with an altered expression between normal and obese iPSCs in control as well as those treated with palmitate.

**Fig. 2:**
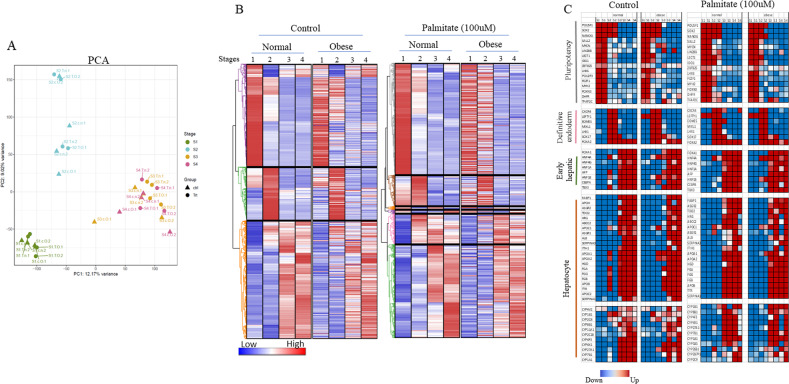
Whole transcriptome analysis of control and palmitate treated normal and obese iPSCs during in vitro hepatocyte differentiation. **A** Principal Component Analysis (PCA) on expressed genes from four stages of hepatic differentiation in normal and obese iPSCs in control and palmitate treated conditions. **B** Hierarchical clustering analysis and corresponding heat map of differentially expressed genes (DEGs) in control and palmitate (100 μM) treated normal and obese iPSCs from four stages of hepatic differentiation. Hierarchical clustering was performed using Euclidean distance to visualize the expression of genes across groups that were significant in at least one inter-stage comparison with FDR < 5%. **C** Heat map of select DEGs from four stages, pluripotency (S1), definitive endoderm (S2), early hepatocyte (S3), and hepatocyte (S4) stages.

Principal component analysis (PCA) shows stage progression, i.e., undifferentiated iPSCs on day 0 of hepatic differentiation (S1 cluster), which is followed by S2 (day 6 of hepatocyte differentiation) along the axis and so on. This suggests that gene clusters from both normal and obese and treated vs nontreated genes have similar gene expression profiles on their specific stages of hepatic differentiation and are different from day 0 (undifferentiated) to day 6 (endoderm) to day 13 & day 17 (hepatoblasts and hepatocytes). However, there was no clear separation between S3 (day 13 of hepatic differentiation) and S4 (day 17 of hepatic differentiation), and it seems these two stages of hepatic differentiation are not too different from each other (Fig. 2A). Globally differentially expressed genes (DEGs) were identified as either up or downregulated with a p-value of less than 0.05 and a false discovery rate (FDR) <5%. Treatment comparison groups revealed specific gene clusters with upregulated and downregulated genes represented specific stages of hepatic differentiation (Fig. 2B).

Hierarchical clustering was performed for a total of 10,374 genes, which have shown altered expression in at least one stage out of 4-stages analyzed in both normal and obese iPSCs (Fig. 2B) in nontreated conditions (N vs O-un) and 9,478 genes in palmitate treated condition (N vs O-palm) (Fig. 2B). Three major gene clusters emerged from the analysis of the first comparison group (N vs O-un), cluster-1 (3888 genes), cluster-2 (2017 genes), and cluster-3 (4469 genes). Whereas the second comparison group (N vs O-palm) resulted in four major clusters and two minor gene clusters, cluster-1 (3864 genes), cluster-2 (1067 genes), cluster-3 (122 genes), cluster-4 (136 genes), cluster-5 (1056 genes) and cluster-6 (3233 genes.

The genes from cluster-1 from both comparison groups were highly expressed in undifferentiated cells (Stage-1, day 0 of hepatocyte differentiation). Once the cells started to differentiate, their expression was reduced in all other three stages (day 6, day 13, and day 17). The genes in this cluster included major pluripotency-related genes such as POU5F1, SOX2, NANOG, LIN28, MYCN, SALL2, among others (Fig. 2C). It was noted that the expression of three pluripotency genes, POU5F1, NANOG, and SOX2, continued to stage-2 (endoderm stage) of differentiation in contrast to other pluripotency related genes, possibly due to ectopic expression of these genes used to induce pluripotency. The genes of cluster-2 were overexpressed specifically on stage2 (day 6 of hepatocyte differentiation) and were enriched in the endoderm-specific genes such as EOMES, SOX17, and FOXA2, suggesting that both types of iPSCs have acquired endoderm fate on the day 6 of hepatocyte differentiation. No major difference in the expression of endoderm-specific markers between normal or obese iPSCs nor the treatment with palmitate was noted, suggesting that obese genotype or palmitate do not affect endoderm fate acquisition. The genes of cluster-3 in the N vs O-un groups and clusters-3,4,5 & 6 of the N vs O-palm groups were enriched in hepatoblasts and hepatocyte-specific markers. The expression of hepatoblast markers such as FOXA1, HNF4α, HNF4γ, AFP, among others, commences on day 13. In contrast, hepatocyte-specific genes such as ALB, SERPINA3, APOB, and various CYP genes express on both day 13 and day 17 of differentiation. The expression of other CYP genes commences only on day 17, suggesting hepatocyte maturation. Again, no major difference in the expression of these genes between normal and obese iPSCs in the nontreated cells or due to palmitate treatment was noted, suggesting that hepatocyte fate acquisition is not affected due to obesogenic genotype or saturated fatty acid exposure during differentiation (Fig. 2C).

### Gene expression changes and pathway enrichment

Next, we performed differential gene expression analysis using the DESeq2 tool to identify genes differentially expressed (DEGs) between normal (N) and obese (O) iPSCs in the solvent treated (C) and palmitate treated (T) cells from all four stages of hepatocyte differentiation. We made four different types of comparisons to identify DEGs from each stage (i) C–O vs N (ii) T–O vs N (iii) N–T vs C (iv) O–T vs C.

**Fig. 3:**
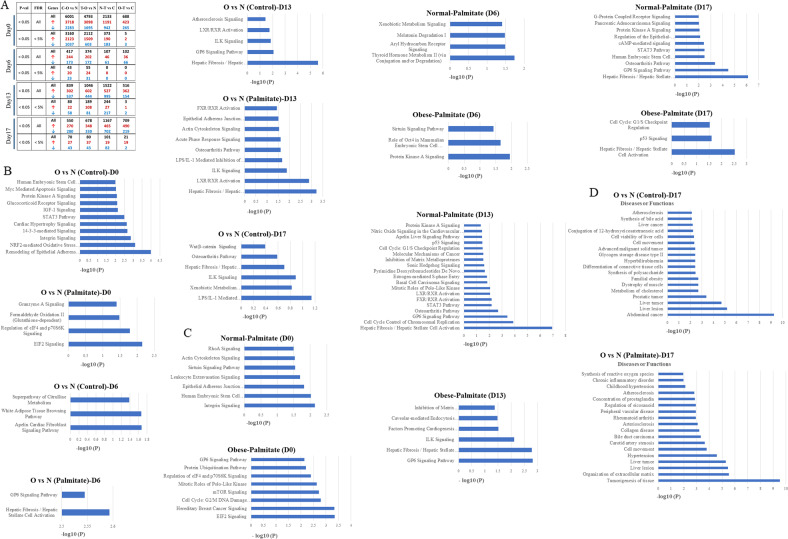
Pathway analysis and disease association of DEGs in control (C) and palmitate treated (T), normal (N)-iPSCs, and obese (O)-iPSCs during in vitro hepatocyte differentiation. **A** The number of genes up/downregulated in four comparison groups (O vs N in C and T iPSCs and T vs C in N-iPSCs and O-iPSCs with different *p* values and false discovery rate (FDR). **B**, **C** Ingenuity Pathway Analysis was performed on the list of genes significantly affected (*p* value ≤ 0.5) in comparison groups **B** O vs N in C and T-iPSCs and **C** T vs C in N and O-iPSCs on D0, D6, D13 and D17 of hepatic differentiation. **D** Disease/Function association analysis on the list of genes affected significantly (*p* value ≤ 0.5) in C and T, normal (N) and obese (O)-iPSCs on D17 of hepatocyte differentiation.

We found a large number of genes (4000–6000 with a *p* ≥ 0.5) were differentially expressed in obese iPSCs compared to normal iPSCs in the control and palmitate treated undifferentiated cells (day 0, (i) C–O vs N (ii) T–O vs N) (Fig. 3A). Moreover, a comparison between normal and obese iPSCs treated with palmitate compared to control ((iii) N–T vs C (iv) O–T vs C) shows a lesser number of DEGs in obese iPSCs compared to normal, suggesting the possibility of a compensatory response in obese iPSCs exposed to excess fat. The numbers of DEGs at the other three stages of hepatocyte differentiation (day 6, day 13, and day 17) were less compared to day 0; however, there were consistently lesser numbers of DEGs in obese iPSCs compared to normal in palmitate treatment (iii) N–T vs C (iv) O–T vs C) at all stages of hepatocyte differentiation analyzed (Fig. 3A).

Furthermore, ingenuity pathway analysis was performed on the list of genes significantly altered in their expression (*P* < 0.5) in all four comparison groups, (i) C–O vs N (ii) T–O vs N (iii) N–T vs C (iv) O–T vs C on all four stages of differentiation (Supplementary files 1–16). On day 0, we found that pathways such as protein kinase A signaling, Glucocorticoid receptor signaling, integrin signaling, and NRF2 mediated oxidative stress response showed the largest and most prominent numbers of genes affected among other pathways in obese iPSCs compared to normal. Whereas the same cells, when treated with palmitate, showed fewer pathway enrichment and included EIF2 signaling as the most affected. On day 6 of hepatocyte differentiation (DE stage), we saw the least DEGs and pathways enriched out of all four stages, and this included Hepatic fibrosis/Hepatic stellate cell activation enriched in obese iPSCs treated with palmitate compared to normal iPSCs. Interestingly, both on days13 and 17 of hepatocyte differentiation (hepatoblasts and hepatocyte stages, respectively), we found Hepatic fibrosis/Hepatic stellate cell activation among other affected pathways, enriched in obese iPSCs in both control and palmitate treated cells compared to normal on day 13 and nontreated obese iPSCs on day 17. However, no major pathway enrichment was observed on day 17 in palmitate-treated obese iPSCs. In addition to the Hepatic fibrosis/Hepatic stellate cell activation pathway, we also found LXR/RXR activation pathway enriched on day 13 in both control and palmitate-treated obese iPSCs (Fig. 3B).

We then compared pathway enrichment of DEGs in normal and obese iPSCs treated with palmitate compared to nontreated control cells to identify the effect of palmitate between normal and obese iPSCs on all four stages of hepatocyte differentiation (Fig. 3C). In addition to several pathways enriched in these cells due to palmitate treatment, we again found Hepatic fibrosis/Hepatic stellate cell activation was enriched in normal iPSCs treated with palmitate on day 13 and day 17. LXR/RXR activation, FXR/RXR activation, and STAT3 pathways were also enriched mainly in normal iPSCs treated with palmitate on both days13 and 17, whereas the numbers of pathways enriched in obese iPSCs treated with palmitate were much less compared to normal iPSCs correlating with lesser DEGs in these cells.

We also analyzed disease associations with DEGs on day 17 in obese iPSCs both control and treated with palmitate compared to normal iPSCs (Fig. 3D). Remarkably, obese iPSCs compared to normal showed several pathologies such as familial obesity, liver lesion, liver tumor, and hyperbilirubinemia, among others. Obese iPSCs treated with palmitate also showed liver cancer and tumorigenesis of tissue enriched among other pathologies.

Thus, we conclude that an obesogenic genotype is associated with pathological transcriptome expression during hepatogenesis, whereas exposure to excess fat on this genome could alleviate or compensate for some gene expression defects. We also presume that LXR/RXR and FRX/RXR pathways could be involved in disease associations identified in obese iPSCs.

### Obese iPSCs exhibit increased lipid accumulation and gene expression changes associated with hepatic steatosis

**Fig. 4:**
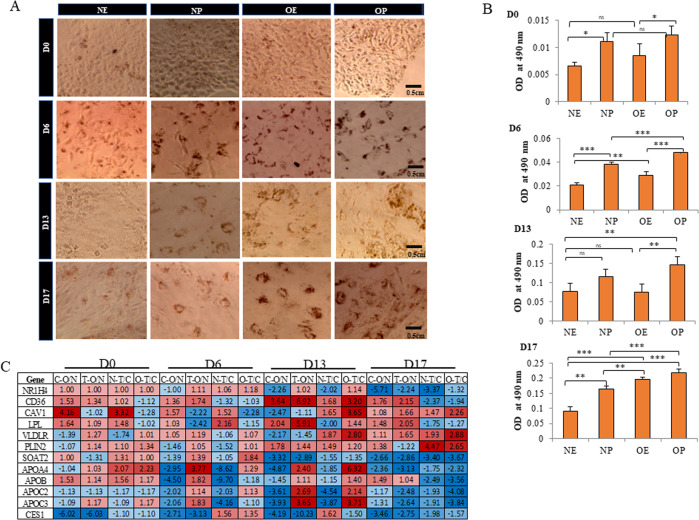
Obese iPSCs exhibit increased oil uptake and upregulation of genes associated with lipid accumulation. **A** Oil Red O staining showing lipid accumulation on day 0, day 6, day 13, and day 17 in (**i**) NE-normal iPSC-derived, ethanol-treated (**ii**) NP**-**normal iPSC-derived palmitate (**iii**) OE**-** Obese iPSC-derived, ethanol-treated and (**iv**) OP-Obese iPSC-derived, palmitate treated differentiating cells. Images were captured using the EVOS XL-Core phase-contrast microscope at ×20 magnification. Scale bar = 0.5 centimeters. **B** Bar diagrams representing the quantitation of fat uptake on (**i**) day 0, (**ii**) day 6, (**iii**) day 13, and (**iv**) day 17 in Normal Ethanol (NE), Normal Palmitate (NP), Obese Ethanol (OE) and Obese Palmitate (OP) treated differentiating cells. The bars represent the mean + SD of three biological replicates. ‘*’ represent the statistical significance of the data. *P < 0.05, **P < 0.01 and **P < 0.001. ns not significant. **C** Heat map of select genes involved in lipid uptake, identified from the list of significantly affected (*p* < 0.5) DEGs in control (C) and palmitate treated (T), normal (N)-iPSCs, and obese (O)-iPSCs during in vitro hepatocyte differentiation from all four stages of hepatic differentiation.

Hepatic Fibrosis/Hepatic Stellate Cell Activation and liver tumorigenesis were major pathways enriched in obese iPSCs compared to normal and in both normal and obese iPSCs when treated with palmitate. Lipid accumulation and hepatic steatosis are the main factors leading to liver fibrosis and hepatocellular carcinoma. Therefore, we first analyzed fat accumulation in obese and normal iPSCs in control and treated with palmitate by Oil red O staining. Normal iPSCs-derived day 6 and day 13 cells challenged with palmitic acid showed increased levels of fat uptake similar to the palmitic acid-treated obese iPSCs-derived cells in the corresponding stages (Fig. 4A, B). However, lipid droplets stored in obese iPSCs were higher than palmitate-treated normal hepatocytes on day 17 of hepatic differentiation. Although normal iPSCs-derived nontreated hepatocytes stain positive for Oil Red O stain, which indicates that the in vitro-derived cells are functional, the amount of lipids stored in these cells is lesser than all other three conditions. At all four stages of differentiation, stored lipid droplets were the highest in obese iPSCs-derived cells treated with palmitate, indicating the increased risk of the perturbed metabolic environment in genetically obese individuals (Fig. 4A, B).

The role of LXR and FXR pathways in hepatic lipid homeostasis is well studied [10]. Since we found LXR and FXR pathways enriched in obese iPSCs and normal and obese iPSCs when treated with palmitate, it is possible that affected genes regulated by these nuclear hormone receptors could have led to this increased lipid accumulation. Although the expression of LXRα (NR1H2) and LXRβ (NR1H3) did not change transcriptionally in obese iPSCs compared to normal or with palmitate treatment; we did see the expression of FXR (NR1H4) being downregulated in obese iPSCs compared to normal as well as in normal iPSCs treated with palmitate on day 17 (Fig. 4C). FXR regulates several hepatocyte functions independently or along with LXR, such as lipoprotein assembly and secretion by regulating the expression of lipid droplet associated proteins, hepatic lipid uptake, de novo lipogenesis, and fatty acid oxidation [10, 11]. When checked, we found the expression of many genes belonging to the above-mentioned functional categories affected in our RNA-seq data (Fig. 4C and Supplementary files 1–16). More specifically, FXR, CD36, CAV1, LPL, VLDLR, PLIN2, SOAT2, APOA4, APOB, APOC2, and APOC3 were altered, especially on day 13 and day 17 in obese iPSCs, and palmitate treated iPSCs. We also found the expression of carboxylesterase 1 (CES1) reduced in obese iPSCs in both untreated control and palmitate treated conditions on all four stages of hepatocyte differentiation compared to normal iPSCs suggesting an inherently reduced CES1 expression in obese iPSCs. It is important to note that FXR controls CES1 expression and regulates fatty acid oxidation; however, hepatic CES1 was essential for both normal and FXR-controlled lipid homeostasis [12]. A recent study suggested that Copy number losses (<2) of CES1 contribute to susceptibility to NAFLD in the Chinese Han population [13]. CES1 expresses at a high level in normal hepatocytes where it hydrolyzes various esters, including cholesterol esters and triglycerides. A reduced expression of CES1 observed in obese iPSCs compared to normal iPSCs could have led to reduced cholesterol and triglyceride breakdown resulting in lipid accumulation observed in obese iPSCs (Fig. 4A, B).

Moreover, previous studies have identified several TFs and regulatory RNAs that regulate hepatocytes’ metabolic homeostasis, and their dysregulation is responsible for hepatic pathologies. Ingenuity pathway analysis led us to identify several such TFs differentially expressed in obese iPSCs and in iPSCs treated with palmitate (Fig. S1A). Many of the identified TFs such as PGC1α, JUN, FOXA1, SMAD3, RUNX, GATA2, among others, are well-known regulators of hepatic metabolism and pathology [14, 15]. We also found the expression of KAT2B (PCAF), a histone acetyltransferase, to be upregulated, specifically in obese iPSCs compared to normal iPSCs in both nontreated and palmitate treated cells. The role of PCAF in hepatic metabolic homeostasis and metabolic syndrome is well known [16, 17], and its differential expression in obese iPSCs points towards epigenetic dysregulations.

We also identified several lncRNAs differentially expressed in obese iPSCs compared to normal iPSCs in nontreated and palmitate-treated cells. Figure S1B shows ten such differentially expressed lncRNAs which are already reported to be associated with pathologies; for example, NEAT1 and MALAT1 [18, 19]. We found the expression of lncRNA, TPTEP1 upregulated in both nontreated and palmitate-treated obese iPSCs compared to normal on day 6, day 13, and day 17 of hepatocyte differentiation (Fig. S1B). Interestingly this lncRNA is found to inhibit hepatocellular carcinoma progression [20] and inhibits the proliferation of non-small cell lung cancer cells [21]. The most highly upregulated lncRNA in obese iPSCs, LINC00662, is recently reported to promote hepatocellular carcinoma progression via altering genomic methylation profiles [22]. Importantly, this lncRNA was highly upregulated at all four stages of hepatocyte differentiation in obese iPSCs in nontreated, and palmitate treated cells, suggesting an inherent higher expression in obese iPSCs than in normal cells.

### Key transcriptional gene dysregulations associated with hepatic steatosis and liver fibrosis

**Fig. 5:**
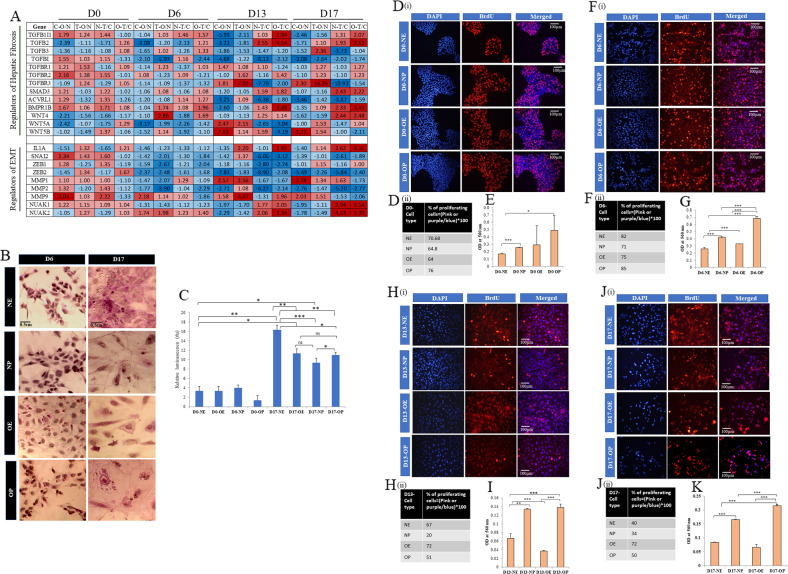
Functional characterization and cell proliferation reveal changes between normal and obese iPSCs. **A** Heat map of select genes known to be associated with hepatic fibrosis and epithelial to mesenchymal transition (EMT), identified from the list of significantly affected (*p* < 0.5) DEGs in control (C) and palmitate treated (T), normal (N)-iPSCs and obese (O)-iPSCs during in vitro hepatocyte differentiation from all four stages of hepatic differentiation. **B** Periodic Acid-Schiff (PAS) staining for Glycogen Storage on day 6 in definitive endodermal cells and day 17 in hepatocytes in (i) Normal Ethanol (NE), (ii) Normal Palmitate (NP), (iii) Obese Ethanol (OE) and (iv) Obese Palmitate (OP) treated differentiating cells. Images were captured using the EVOS XL-Core phase-contrast microscope at ×40 magnification. Scale bar = 0.5 centimeters. **C** Bar plots representing Luciferase-IPA Assay for CYP3A4 activity in iPSCs-derived hepatocytes on day 17 of differentiation. Luminescence was measured using the TECAN InfiniteM200 Pro plate reader. The bars represent Mean+SD from three biological replicates, and * represents statistical significance. *P < 0.05, **P < 0.01 and ***P < 0.001. ns not significant. **D** Cell proliferation and Cytotoxicity Assays. Bromodeoxyuridine (BrdU) immunostaining to assay cell proliferation on day 0, day 6, day 13, and day 17 in Normal Ethanol (NE), Normal Palmitate (NP), Obese Ethanol (OE) and Obese Palmitate (OP) treated differentiating cells. Images were captured using the ×40 objective of Olympus IX70 inverted fluorescence microscope. Scale bar =100 micrometers. The percentage of proliferating cells was calculated using ImageJ and tabulated for **D-ii** pluripotent cells on day 0, **F-ii** definitive endoderm cells on day 6, **H-ii** hepatoblasts on day 13, and **J-ii** hepatocytes on day 17. Cytotoxicity Assay to measure Lactate Dehydrogenase (LDH) release **E** pluripotent cells on day 0, **G** definitive endoderm cells on day 6, **I** hepatoblasts on day 13, and **K** hepatocytes on day 17. Absorbance measured at 560 nm and mean +SD from three replicates is represented as bar plots. ‘*’ represent statistical significance. *P < 0.05, **P < 0.01, and ***P < 0.001. ns not significant.

Activation of TGFβ signaling is found to be associated with liver fibrosis in several previous studies [23]. In correlation with liver fibrosis as one of the major pathways enriched among DEGs in obese and palmitate treated iPSCs, we found several genes of the TGFβ signaling pathway also differentially expressed (Fig. 5A and Supplementary files 1–16) mostly on day 13 and day 17 of hepatic differentiation. In addition, the expression of BMPR1B, ACVRL1, and three genes of the Wnt pathway (WNT4, WNT5A, and WNT5B) were also differentially expressed in obese iPSCs as well as palmitate treated cells. Moreover, similar to the decreased levels of epithelial marker E-CAD in day 17 hepatocytes (Fig. 1H), which is a feature of epithelial to mesenchymal transition (EMT), we also found the expression of other EMT markers such as IL1A, ZEB1, ZEB2, SNAIL MMP2, MMP1, and MMP9 affected mostly in the latter two stages of hepatocyte differentiation (Fig. 5A).

We also compared DEGs of days 13 and 17 against differentially expressed genes from a published RNA-seq data set (GSE126848) of patients with varying degrees of NAFLD [24]. We found 15–34% overlap among genes upregulated in different sets of NAFLD samples vs day 13/day 17 of hepatocyte differentiation (Fig. S2A). Similarly, there was 12–26% overlap among genes that were downregulated (Fig. S2A). When we compared specifically some of the genes which were altered in our RNA-seq data as mentioned above with NAFLD samples, we noticed different patterns. For example, genes, BMPR1B, TGFB2, TGFB1I1, SMAD3, NUAK1, and NUAK2 were downregulated in obese iPSCs in both control and palmitate treated cells compared to normal iPSCs also showed downregulation to varying degrees in NAFLD samples (Fig. S2B). On the contrary, genes LPL, MMP9, APOC2, APOC3, APOA4, ZEB2, TGFBI, MMP2 which were all upregulated in NAFLD samples were also upregulated only in obese iPSCs in both nontreated and palmitate treated cells, particularly on day 13 (Fig. S2B and Supplementary file 17). These results further confirm the pathological nature of obese iPSCs.

To find out whether differential expression of these genes is associated with cell fate changes, we proceeded with functional characterization of normal and obese iPSCs in control and treated with palmitate from all four stages of hepatocyte differentiation. Periodic acid-Schiff (PAS) staining was carried out to confirm that the in vitro differentiated hepatocytes were functional and to compare the levels of glycogen stored in the normal and obese iPSCs-derived hepatocytes under palmitate treated and control conditions. DE cells (day 6) corresponding to each condition were used as a comparison control. In control cells, normal iPSCs-derived hepatocytes stored glycogen abundantly in the cytoplasm compared to their predecessors on day 6 (Fig. 5B). In the presence of excess saturated fatty acids, hepatocytes derived from normal iPSCs showed a remarkable reduction in stored glycogen, which was almost similar to the levels on day 6. Although they showed the characteristic polygonal morphology of functional hepatocytes, the cytoplasm appeared pale brown. Intriguingly, at some sites, in addition to the hematoxylin stain within the nuclei, abnormal pink staining was observed, suggesting the abnormal localization of glycogen within the nuclei of these cells (Fig. 5B). The hepatocytes derived from obese iPSCs under palmitate treated and control conditions displayed the polygonal morphology of hepatocytes and pink-colored cytoplasm, indicating the presence of stored glycogen. The intensity of PAS-stained cells was higher in palmitate-treated obese hepatocytes compared to their nontreated control counterparts. Moreover, abnormal pink staining of the nuclei was observed in these hepatocytes, similar to the fatty acid-challenged conditions in normal hepatocytes (Fig. 5B).

Luciferase-IPA assay was carried out to measure the liver-specific CYP3A4 activity as a confirmatory test for the iPSCs-derived hepatocytes. The relative luminescence was measured and plotted to compare the CYP3A4 activity of the hepatocytes generated under all four conditions of differentiation. A statistically significant increase in CYP3A4 activity was observed on day 17 in all four conditions compared to their counterparts on D0, thereby confirming that the hepatocytes are functional concerning drug or toxin metabolism. As expected, the nontreated normal hepatocytes exhibited better CYP3A4 activity than the fatty acid-treated hepatocytes and obese iPSCs-derived hepatocytes maintained in the presence or absence of high fat. Within the last three conditions, we did not observe a remarkable difference in CYP3A4 activity that was statistically significant (Fig. 5C).

BrdU staining and lactate dehydrogenase (LDH) assays were performed to compare cell proliferation and cytotoxicity at all four stages and conditions of hepatic differentiation of the iPSCs. The undifferentiated pluripotent iPSCs showed 65–75% proliferation, and cytotoxicity was higher in the palmitate-treated iPSCs than in nontreated cells (Fig. 5D–i&ii). Interestingly, palmitate treatment favored proliferation in obese iPSCs compared to the normal iPSCs. At the same time, cytotoxicity due to palmitate treatment was higher in both normal and obese iPSCs (Fig. 5E). As the cells progressed to the Definitive Endoderm (DE) stage, albeit higher cell proliferation on day 6 compared to day 0 (Fig. 5F–i&ii), cytotoxicity was higher than in undifferentiated cells, particularly in palmitate-treated Obese iPSCs-derived cells, as is evident by the lesser number of DAPI-stained cells (Fig. 5G).

Although cytotoxicity was lesser in the iPSCs-derived hepatoblasts than in the DE stage, a drastic reduction in cell proliferation was observed in palmitate-treated normal iPSCs-derived hepatoblasts (Fig. 5H, i&ii). This may be due to palmitate-induced cytotoxicity coupled with selection pressure of differentiation (Fig. 5I). On day 13 of differentiation, cytotoxicity was lesser in nontreated obese hepatoblasts compared to their normal counterparts but was increased in palmitate-treated obese hepatocytes (Fig. 5I).

At the last stage of differentiation, the polygonal hepatocytes were larger than the oval-shaped hepatoblasts and hence, covered more surface area on the cultured wells. Hence per field, the number of DAPI-stained hepatocytes is lesser compared to the hepatoblasts. In treated conditions, higher cytotoxicity and lesser cell proliferation were observed, with the least number of BrdU stained cells in normal iPSCs-derived hepatocytes and the highest cytotoxicity in obese iPSCs-derived hepatocytes (Fig. 5J–i&ii). As expected, normal iPSCs-derived hepatocytes were less proliferative than on day 13. However, surprisingly, the obese iPSCs-derived hepatocytes remained as proliferative as their predecessors even in the last stage of differentiation.

Therefore, palmitate treatment led to cell death in both normal and obese iPSCs. No major difference in cell proliferation was observed between normal and obese iPSCs between control, and palmitate treated conditions except on day 13, where palmitate treatment significantly reduced cell proliferation in normal iPSCs. Interestingly, obese iPSCs-derived hepatoblasts and hepatocytes were more proliferative than normal iPSCs-derived cells without palmitate treatment. This further suggests that obese iPSCs show increased cell proliferation in the early stages of hepatocyte differentiation and a transcriptome profile that is pathological in nature.

### Palmitate upregulates PCAF expression and histone H3K27Ac levels with the highest response in obese iPSCs

**Fig. 6:**
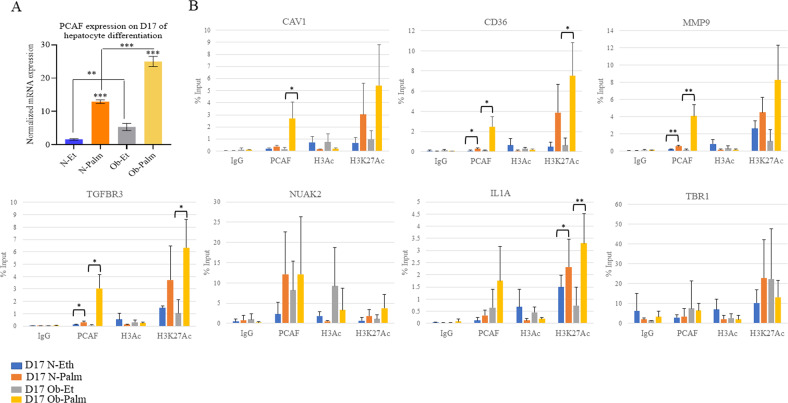
Effect of palmitate treatment and obese genotype on PCAF expression and histone acetylation. **A** mRNA expression analysis of PCAF gene by RT-qPCR in normal (N) and obese (Ob) iPSCs treated with solvent control (Ethanol) and 100 μM palmitate (Palm) at day 17 of hepatocyte differentiation using gene-specific primers. The expression of the β-ACTIN gene was used to normalize the expression. The data are represented as mean ± standard deviation. **p ≤ 0.01, ***p ≤ 0.001 (two-tailed unpaired Student’s *t* test). The data are representative of three independent biological replicates. **B** Normal and obese iPSCs-derived hepatocytes (D17) were used to perform ChIP assay for PCAF, H3Ac, and H3K27Ac using ChIP grade antibodies. ChIP with IgG was used as the negative control. ChIP’d DNA was analyzed by real-time qPCR for the promoter regions of CAV1, CD36, MMP9, TGFBR3, NUAK2, IL1A, and TBR1 genes. Data represent the mean of three biological replicates ± SD. Statistical analyses (unpaired student’s *t* test) were made between control (Et) and palmitate (Palm) treated samples using GraphPad Prism Software. Asterisks represent differences being significant 1098 (*p < 0.05, **p < 0.01, ***p < 0.001).

Epigenetic dysregulations resulting from chronic metabolic perturbations are known to be associated with pathophysiological development [25, 26]. Identifying epigenetic biomarkers in individuals with a genetic predisposition to obesity could be considered to avoid further exacerbation due to gene-environment interactions [27]. As mentioned above, we found the expression of KAT2B (PCAF), a histone acetyltransferase, upregulated after palmitate treatment in both normal and obese iPSCs. We validated the results of RNA-seq on PCAF expression through RT-qPCR analysis. We found the PCAF mRNA levels to be higher in obese iPSCs than normal iPSCs, and palmitate treatment further upregulates PCAF expression where obese iPSCs treated with palmitate showed the highest expression (Fig. 6A). In order to understand whether PCAF upregulation could have affected gene expression in control and palmitate treated normal and obese iPSCs, we performed Chromatin immunoprecipitation (ChIP) for PCAF, total H3Ac, and H3K27Ac, which is commonly present on promoters and enhancers and performed RT-qPCR for the promoters of six genes (CAV1, MMP9, CD36, TGFBR3, NUAK2, IL1A) which have shown upregulation in obese iPSCs compared to control and after palmitate treatment. As a control, we also amplified the promoter of TBR1, which normally did not express in hepatocytes and did not show altered expression in the RNA-seq experiment. ChIP for IgG was used as the negative control. We found the recruitment of PCAF to be higher in palmitate-treated normal iPSCs compared to control, whereas this PCAF recruitment was further increased in obese iPSCs in palmitate-treated conditions compared to normal iPSCs. Interestingly, the levels of histone, H3K27Ac, were similarly elevated in palmitate-treated cells, being highest in obese iPSCs (Fig. 6B). However, the levels of total H3Ac were not increased, rather, a reduced enrichment of total H3Ac was observed in palmitate treated normal and obese iPSCs compared to control for all of the genes analyzed except TBR1. This suggests that elevated histone acetylation is specific for H3K27Ac. These results led us to conclude that increased PCAF expression in obese and palmitate treated iPSCs compared to normal iPSCs could have led to elevated H3K27acetylation on the promoters of genes with increased mRNA expression. Therefore, increased histone acetylation could be one epigenetic mechanism responsible for pathological features of normal palmitate-treated iPSCs as well as obese iPSCs in both nontreated and palmitate-treated conditions.

### Pharmacological intervension reduces lipid accumulation in obese iPSCs

**Fig. 7:**
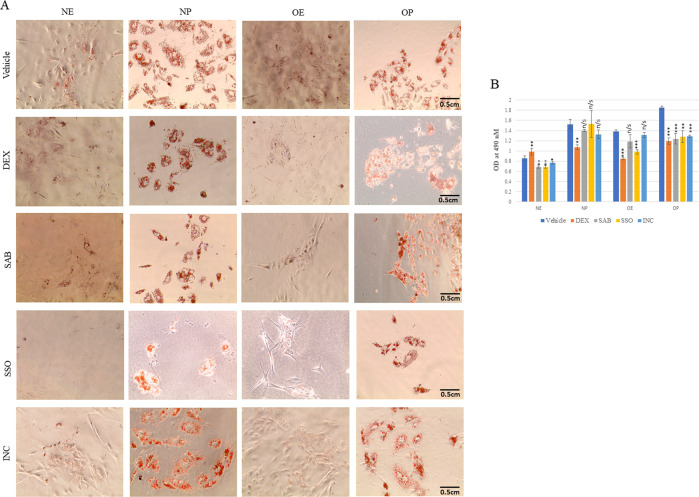
Pharmacological inhibition of CD36 and CAV1 reduces lipid accumulation in obese iPSCs-derived hepatocytes. **A** Oil Red O staining showing fat uptake in hepatocytes from (i) NE-normal iPSC-derived, ethanol-treated (ii) NP-normal iPSC-derived palmitate treated (iii) OE- Obese iPSC-derived, ethanol-treated and (iv) OP-Obese iPSC-derived, palmitate treated. On day 17 of hepatocyte differentiation, the cells were treated with dexamethasone (DEX, 10 μM), Salvianolic Acid B (SAB), 200 μM), Sulfosuccinimidyl oleate sodium (SSO, 200 μM), Incadronate (INC, 200 μM) and vehicle (solvent control) for 72 h. Images were captured using the EVOS XL-Core phase-contrast microscope at ×20 magnification. Scale bar = 0.5 centimeters. **B** Bar diagrams representing the quantitation of fat uptake as described in **A**. The bars represent the mean ± SD of three replicates. ‘*’ represent the statistical significance of the data. *P < 0.05, **P < 0.01, and ***P < 0.001. ns not significant.

In order to test whether increased lipid accumulation in obese iPSCs possibly due to upregulated expression of genes such as CD36 and CAV1 could be inhibited due to pharmacological targeting of these genes. We used two known inhibitors of CD36, salvionolic acid B (SAB) and sulfosuccinimidyl oleate (SSO) [28–30]. Normal and obese iPSCs were differentiated into hepatocytes treated with palmitate and solvent control for seventeen days. Afterwards, the cells were treated with SAB (200 µM) and SOS (200 µM) for 72 h. Similarly, we also used incadronate (INC), an inhibitor of CAV1 [31] and dexamethasone (DEX), known to inhibit CES1 [32] which could result in increased lipid accumulation. We found that both CD36 inhibitors, SAB and SSO led to significantly reduced lipid accumulation in normal (solvent treated) and obese (palmitate treated) iPSCs, whereas, only SSO reduced lipid levels in solvent treated obese iPSCs. SAB and SSO did not reduce lipid in normal iPSCs-derived hepatocytes when treated with palmitate. CAV1 inhibitor, INC also reduced lipid in normal (solvent treated) and obese (palmitate treated) iPSCs whereas no effect was observed in other conditions (Fig. 7A, B). Since the expression of CES1 was found to be significantly reduced in obese iPSCs which could lead to increased lipid accumulation, we treated these cells with rifampicin which has been shown to induce the expression of CES1 [32] whereas dexamethasone (DEX) has an opposite effect. Rifampicin-treated cells did not survive due to excessive cytotoxicity however, as expected, DEX led to a subtle but significant increase in lipid accumulation in normal solvent-treated iPSCs. However, normal palmitate treated and both solvent and palmitate treated obese iPSCs showed reduced lipid accumulation (Fig. 7A, B). This was not completely unexpected as it was shown previously that treatment of primary rat hepatocytes with DEX increased lipid content but further treatment with palmiate either decreased or had no effect on intrahepatocellular triacylglycerol levels [33]. Overall, these results led us to conclude that pharmacological inhibition of CD36 and CAV1 reduce lipid levels in obese iPSCs-derived hepatocytes and could be used preemptively to avoid excessive lipid accumulation and associated pathologies.

## Discussion

Liver diseases associated with obesity and metabolic syndromes such as non-alcoholic fatty liver disease (NAFLD), liver cirrhosis, and hepatocellular carcinoma [34] have increased considerably in the past few decades [35, 36]. Obesity and metabolic syndrome have a genetic predisposition as several genetic variants are found to be associated with obesity and metabolic syndrome [37–39]. At the same time, environmental factors such as unhealthy dietary habits and sedentary lifestyles are also major contributors to obesity and related pathologies [40, 41]. Therefore, the pathology of obesity and associated NAFLD could be a combination of genetic predisposition superimposed on an unhealthy dietary habit leading to pathological development [42–44]. Moreover, the developmental origin of metabolic disease hypothesis proposes that in-utero or early life exposure to a perturbed metabolic environment such as maternal obesity could influence the susceptibility to metabolic disease later in life [45–47]. Therefore, it is possible that such individuals acquire a gene expression profile that is pathological in nature due to subtle changes in gene expression early on during development, which could be responsible for pathologies such as NAFLD later in life.

Hepatic epithelial cells, mainly hepatocytes, are susceptible to pathologies of non-alcoholic fatty liver disease and liver cirrhosis. Therefore, to understand the relationship between an obesogenic genotype combined with an abnormal metabolic environment such as high free fatty acids, we have used hiPSCs from super-obese and normal subjects [4] and differentiated them into hepatocytes in cell culture in the presence and absence of palmitate, a saturated fatty acid. We then performed RNA-seq on cells from four key stages of hepatogenesis to identify differences in gene expression between normal and obese iPSCs and the effect of their exposure to palmitate. Among hundreds of genes that were differentially expressed, we found several genes which were remarkable for their known involvement in hepatocyte pathologies. For example, the expression of fatty acid translocase CD36 was upregulated on days 13 and 17 of hepatocyte differentiation in the obese iPSCs compared to normal, and treatment with palmitate further increased its expression. The expression of caveolae protein, CAV1 showed subtle upregulation in normal iPSCs when treated with palmitate, whereas this upregulation was more pronounced in obese iPSCs on both day 13 and day 17. The expression of lipid droplet binding protein PLIN2 was upregulated after palmitate treatment, specifically on day 17 in both normal and obese iPSCs.

Interestingly, apolipoproteins, APOA4, APOB, APOC2, and APOC3 showed upregulation only in obese iPSCs in the presence of palmitate. These results support the possibility of an increased import and storage of lipids in normal iPSCs when treated with palmitate but in obese iPSCs even in the absence of palmitate treatment; suggesting an inherent property of lipid accumulation in obese iPSCs. The increased lipid accumulation observed in obese iPSCs may also have resulted from decreased expression of carboxylesterase 1 (CES1) gene, which was observed specifically in obese iPSCs in both control and palmitate treated conditions at all four stages of hepatocyte differentiation. Hepatic CES1 has triglyceride hydrolase activity and is known to be involved in hepatic lipid homeostasis [12]. Knockdown of hepatic CES1 in mice increased hepatic triglyceride and plasma cholesterol levels, possibly from the triglyceride hydrolase activity of CES1 [12]. Therefore, the increased lipid accumulation observed in obese iPSCs in this study could have resulted from decreased triglyceride breakdown in these cells. Interestingly, pharmacological inhibition of both CAV1 and CD36 reduced lipid accumulation in both normal and obese iPSCs with the effect being most prominent in obese iPSCs when treated with palmitate.

The pathogenesis of NAFLD is a two-step process where lipid accumulation in the hepatocytes is the first step that could lead to hepatic injury, inflammation, and fibrosis in the next step [48]. Transforming growth factor-beta (TGFβ) is considered an inducer of fibrosis development. The expression of TGFβ was reported to increase markedly in several kinds of tissue cells before fibrosis, including in the cirrhotic liver [49, 50]. The expression of TGFβ is also associated with morphologic alterations such as epithelial to mesenchymal transition (EMT) in hepatocytes, and during liver fibrosis, EMT itself may play a relevant role in the appearance of a profibrotic fibroblast phenotype [51, 52]. SMAD3 is considered a crucial inducer of the fibrogenic program, and interestingly we found expression of SMAD3 upregulated on days 13 and 17 of hepatocyte differentiation in both normal and obese iPSCs when treated with palmitate. In addition, the expression of TGFB1I1, TGFB2, and BMPR1B were also similarly upregulated, whereas the expression of TGFBR3 was upregulated in obese iPSCs only, which was further increased several folds due to palmitate treatment on day 13 and 17. In addition, the expression of WNT5A and WNT5B was also up in obese iPSCs. These results suggest the activation of the TGFβ pathway. Moreover, other EMT inducers such as IL1A, MMP1, MMP9, NUAK1, and NUAK2 were also upregulated on days 13 and 17. In support of these changes, we observed decreased levels of epithelial marker E-CAD in day 17 hepatocytes.

We also found the expression of PCAF (KAT2B), a histone methyltransferase, to be upregulated in obese iPSCs compared to normal and due to palmitate treatment. Although the mRNA levels of PCAF were upregulated due to palmitate treatment, nontreated obese iPSCs had higher PCAF mRNA expression than normal iPSCs suggesting an inherently increased PCAF expression due to obesity-prone genotype. Therefore, we decided to find out whether epigenetic modulations could have played a role in gene expression changes observed through PCAF. The role of PCAF in the regulation of effectors of the TGFβ pathway has been reported in previous studies [53, 54]. Moreover, the role of histone acetylation in the pathology of obesity and NAFLD has also been reported before [55–57]. Indeed PCAF recruitment was higher in palmitate-treated normal iPSCs compared to nontreated cells, and obese iPSCs showed the highest PCAF recruitment on several gene promoters, which have shown upregulation in RNA-seq. This increased PCAF recruitment was followed by increased H3K27acetylation on these promoters, whereas total H3Ac levels remained unchanged or were reduced due to palmitate. Histone H3K27Ac is associated with increased transcription on both promoters and enhancers. Moreover, high-fat diet (HFD) fed mice showed increased H3K27Ac levels in the pancreas on genes upregulated in a genome-wide study in mice, suggesting that high-fat modulated gene expression through H3K27acetylation [55] similar to what we have observed due to palmitate treatment of our iPSCs-derived hepatocytes. Future studies involving genome-wide ChIP-sequencing for H3K27Ac, PCAF, and total H3Ac in normal and obese iPSCs will reveal genome-wide effects of this histone modification on promoters as well as enhancers in causing gene expression changes and pathological development.

Taken together, these results have led to the identification of pathological cellular and molecular signatures due to an obesogenic genotype superimposed on a perturbed metabolic environment compared to a non-obese genotype. These results have clearly shown a pathological transcriptome in obese iPSCs and further exacerbation or alleviation due to excess fatty acid exposure. Further studies are needed to identify the transcriptional regulatory mechanisms of transcriptome dysregulation observed in obese iPSCs. In this regard, the role of differentially expressed TFs and lncRNAs, which we have identified here, and chromatin regulator PCAF mediated histone acetylation need to be investigated in future genome-wide studies.

## Materials and methods

### In vitro differentiation of human iPSCs derived from normal and obese individuals to hepatocyte-like cells

Human iPSC lines derived from reprogrammed fibroblasts of normal and obese subjects were purchased from Cedars-Sinai Board of Governors Regenerative Medicine Institute, California. Normal (CS02iCTR-NTn1 and CS03iCTR-NTn1) and obese (CS02iOBS-n4 and CS03iOBS-n3) iPSC lines were maintained on corning Matrigel hESC qualified matrix (Cat. No. 354277, CORNING)-coated plates in mTeSR1 media (Cat. No. 85870, Stem Cell Technologies) supplemented with FGF-basic (Cat No.PHG0026, GIBCO). The iPSC lines were maintained, passaged, and differentiated into hepatocytes as per the three-stage protocol adopted for human ESCs (H9) reported previously [5]. Briefly, the small molecule CHIR99021 (Cat. NO. 4423, Tocris) supplemented Definitive Endoderm (DE) media-generated DE cells (Stage II) on the sixth day of differentiation. DE cells were maintained for 7 days in hepatocyte progenitor media, during which they differentiated into hepatoblasts (Stage III). For the last stage, the hepatic progenitor cells were maintained in Toboul’s hepatocyte maturation media for 4 days, and hepatocyte-like cells were harvested on day 17 of differentiation. During the entire course of differentiation, 100 μM Palmitate (Sigma-Aldrich, Cat. No: CAS 57-10-3) was administered to the normal and obese iPSCs-derived differentiating cells to maintain high levels of saturated fatty acids. Ethanol was used as the control for the fatty acid treatment. For inhibitor treatment, the iPSCs were differentiated into hepatocytes as described above for 17days and then treated with, dexamethasone (10 μM), salvianolic acid B (200 μM), sulfosuccinimidyl oleate sodium (200 μM), and incadronate (200 μM) for 72 h. All cell cultures were regularly tested for mycoplasma contamination using the ‘LookOut Mycoplasma PCR Detection Kit (Sigma-Aldrich MP0035-1KT)’ as per the manufacturer’s instructions.

### Fat uptake and ORO staining

Oil Red Staining was performed as per the protocol described in Lonza’s Protocol Manual [58]. Differentiating cells at all four stages of differentiation were fixed with 3.7% formaldehyde for 10 min at room temperature. Cells were washed with PBS and treated with 60% isopropanol for 5 min. Freshly prepared Oil red O solution was used to stain the fatty acids uptaken by the cells. The cells were washed thoroughly with distilled water and imaged using an EVOS XL-Core phase-contrast microscope at ×20 and ×40 magnification. The stained cells were treated with 100% isopropanol for 5 min, and the OD was measured at 490 nm to quantitate the fatty acid accumulation at each stage of treated and non-treated conditions.

### Functional assays–periodic acid-Schiff staining for glycogen storage and luciferase-IPA assay for CYP3A4 activity

Cells were fixed with 3.7% formaldehyde and permeabilized with Triton X-100. The cells were washed with PBS and stained using the periodic acid-Schiff (PAS) kit (Cat. No. 395B, Sigma). Images were captured at ×20 and ×40 using the EVOS XL-Core phase-contrast microscope.

Non-lytic cell-based assay was carried out to measure CYP3A4 activity, a liver-specific Cytochrome P450, using the P450-Glo^TM^ CYP3A4 Assay (Luciferin- IPA) Kit (Promega, Cat. No. V9001/2). In brief, on day 17 of differentiation, the media is replenished with media containing 3 mM Luciferin-IPA in DMSO and incubated for 30 to 60 min at 37 °C. The luminogenic substrate added to media without cells served as the blank to measure background luminescence. To 25 μl of media transferred to 96-well plates in triplicates, 25 μl detection reagent was added and incubated at room temperature for 20 min. Luminescence was measured using the TECAN InfiniteM200 Pro plate reader.

### Cell proliferation assay

Bromodeoxyuridine (BrdU) detection assay was carried out to detect cell proliferation in treated and nontreated differentiating cells [59].

Differentiating cells at all four-time points were incubated with 0.4 μl/ml of 5-BrdU solution (20 μg/ml in H_2_O) (TOCRIS, Cat#5015) added directly to the growing media, followed by 60 min of incubation at 37 °C. The cells were fixed using 3.7% formaldehyde for 10 min at room temperature and washed with PBS. The fixed cells were treated with 0.6% freshly prepared 30% hydrogen peroxide in PBS (Sigma, Cat # H3410), 2 N HCl for 10 min each, followed by 0.1 M sodium borate solution for 5 min at room temperature. The cells were then blocked, permeabilised and the BrdU stain was immunodetected with mouse Ig2A anti-BrdU (R&D Systems, Cat#MBA7225) and a secondary antibody (goat antimouse Alexa Flour 594 conjugated) as described below.

### Cytotoxicity assay

Lactate Dehydrogenase (LDH) Assay was carried out at all stages of differentiation under treated and nontreated conditions using the CytoTox 96® non-radioactive cytotoxicity assay kit (Promega, Cat. No. G1780) according to the manufacturer’s instructions. Absorbance was measured at 490 nm using the TECAN InfiniteM200 Pro plate reader.

### Immunofluorescence

Cells were maintained on 4-well plates and immuno-stained for stage-specific markers at all four-time points. Cells were fixed with 3.7% formaldehyde for 10 min and incubated in 0.1% Triton X-100 for 10 min. The Triton permeabilized cells were blocked with 1% BSA for 15 min and incubated overnight at 4 °C in respective primary antibodies. The next day, cells were washed with PBS and incubated for 1 h at room temperature with Alexa-conjugated secondary antibodies. Table 1 enlists the antibodies used in the study. The nuclei were stained with DAPI (Millipore, CAS 28718-90-3) for 5 min and imaged at ×40 magnification using a fluorescence microscope (Zeiss, Axiovert 40 CFL).

### Total RNA isolation, cDNA synthesis, and real-time PCR

MasterPure™ RNA purification Kit (Epicenter, Cat.#MCR 85102) was used for total RNA isolation, followed by cDNA synthesis of 1.0 μg of total RNA using SuperScript VILO cDNA Synthesis Kit (Cat.#11,754,050) as per manufacturer’s instructions. RT-qPCR was performed using SYBR® Green Real-Time qPCR Master Mix (ThermoFisher Scientific) in a 10 μl reaction volume. The primer sequences used for RT-qPCR reactions are - PCAF-forward:5′-AGGAAAACCTGTGGTTGAAGG-3′; PCAF-reverse: 5′-CAGTCTTCGTTGAGATGGTGC-3′; 18SrRNA-forward:5′-CTACCACATCCAAGGAAGCA-3′; 18SrRNA-reverse:5′-CTACCACATCCAAGGAAGCA-3′.

### Chromatin immunoprecipitation (ChIP)

ChIP assays were performed as reported previously [60]. Briefly, 2 wells of differentiating cells from a P-24 well plate were pooled after fixing with 1% formaldehyde for 10 min. The cells were washed and lysed in cell lysis buffer. The chromatin was sheared by sonication in diagenode bioruptor for 10 min at 30 sec on/off cycles. The lysate was immunoprecipitated using ChIP grade antibodies to H3Ac (Millipore, Cat. No.: 06-599), PCAF (Cell Signaling Technology, Cat. No.: 3378) and H3K27Ac (ABclonal, Cat. No.: A7253). ChIP with normal IgG (Millipore, Cat # PP64B) was used as the negative control. ChIP’d DNA was recovered and analyzed by RT-qPCR using SYBR® Green Real-Time PCR Master Mix (ThermoFisher Scientific) in a 10 μl reaction volume. Primer sequences used to amplify promoter regions are provided in Supplementary Table 1.

### RNA sequencing

Total RNA was isolated using MasterPure™ RNA Purification Kit from Epicentre (Cat.#MCR 85102) according to the manufacturer’s instructions from normal and obese (control and palmitate treated) iPSCs from days 0, 6, 13, and 17 of hepatocyte differentiation. Libraries were prepared using NEBNext Ultra II RNA Library Prep Kit for Illumina (NEB #E7775) using the manufacturer’s instructions. Briefly, 1 μg of total RNA was used to remove ribosomal RNAs using NEBNext® rRNA Depletion Kit v2 (Human/Mouse/Rat) (E7400L) and purified using AMPure XP beads from Beckman Coulter. Libraries were prepared on the resulting RNA samples, which included RNA fragmentation, cDNA synthesis, adapter ligation, and purification as per instructions from the kit. Adapter ligated cDNA was PCR amplified using NEBNext® Multiplex Oligos for Illumina (E6440S) using unique combinations of i5 and i7 index primers for multiplexing. The libraries were finally purified using AMPure XP beads and proceeded for QC analysis and sequencing. Sequencing was performed at the Genomics Core Facility, The Wistar Institute, Philadelphia, PA on Illumina Next-Generation Sequencer (NextSeq 500), followed by bioinformatics analysis.

RNA-seq data were aligned using bowtie2 [61] algorithm against hg19 human genome version, and RSEM v1.2.12 software [62] was used to estimate read counts and FPKM values using gene information from Ensemble transcriptome version GRCh37.p13. Raw counts were used to estimate the significance of differential expression differences between experimental groups using DESeq2 [63]. Overall gene expression changes were considered significant if passed FDR < 5% unless stated otherwise. Gene set enrichment analysis was done using QIAGEN’s Ingenuity® Pathway Analysis software (IPA®, QIAGEN Redwood City,www.qiagen.com/ingenuity) using “Canonical pathways”, “Diseases &Functions” and “Upstream Regulators” options. Expression heatmaps were generated using DESeq2 normalized count values. The significance of the overlap between stages was tested using Fisher Exact Test. Hierarchical clustering was performed using Euclidean distance to visualize the expression of genes across groups that were significant in at least one inter-stage comparison with FDR < 5%.

### Statistical analysis

hiPSCs-hepatocytes differentiation with and without treatment with palmitate was performed at least 3–4 times. Each experiment was repeated with three biological replicates for each stage of differentiation as indicated except for RNA sequencing, which was performed on two biological replicates. The results shown are mean ± standard deviation (SD). Statistical analyses were made between control, and palmitate-treated samples individually at each time point of differentiation using GraphPad software (unpaired student’s *t*-test) with asterisks representing differences being significant (*P < 0.05, **P < 0.01, ***P < 0.001).

## Supplementary information

Reproducibility checklist

Supplementary text file

Supplementary File-1

Supplementary File-2

Supplementary File-3

Supplementary File-4

Supplementary File-5

Supplementary File-6

Supplementary File-7

Supplementary File-8

Supplementary File-9

Supplementary File-10

Supplementary File-11

Supplementary File-12

Supplementary File-13

Supplementary File-14

Supplementary File-15

Supplementary File-16

Supplementary File 17

## Data Availability

Raw and processed RNA-seq data were deposited to the NCBI GEO database under accession number GSE198391.
