# Supplementary material for: Developmental modeling of hepatogenesis using obese iPSCs-hepatocyte differentiation uncovers pathological features
Source: Cell Death Dis. 2022 Aug 1;13(8):670. doi: 10.1038/s41419-022-05125-9 (PMC9343434; doi:10.1038/s41419-022-05125-9)
Supplement: Supplementary file 2 — Supplementary text file [file 41419_2022_5125_MOESM2_ESM.docx]

**Figure S1.**  Transcription factor (TF) enrichment analysis and long non-coding RNA identification uncovers several key developmental and metabolic genes differentially expressed in obese-iPSCs and after palmitate treatment. (**A**) Heat map of select transcription factor (TF) genes identified by Ingenuity Pathway Analysis (IPA) from the list of significantly affected genes in control (C) and palmitate treated (T), normal (N)-iPSCs and obese (O)-iPSCs during *in-vitro* hepatocyte differentiation. (**B**) Heat map of select long non-coding RNAs differentially expressed in control (C) and palmitate treated (T), obese (O)-iPSCs compared to (N)-iPSCs during *in-vitro* hepatocyte differentiation.

**A**

D0

D6

D13

D17

**B**

D13

D6

D17

D0

**Figure S2.**  Comparison of DEGs from iPSC derived hepatocytes with DEGs from NAFLD patients show overlaps. (**A**) Genes that are significantly up or downregulated on day13 (D-13 or S3) and day17 (D-17 or S4) of hepatocyte differentiation in control (C) and palmitate treated (T), normal (N)-iPSCs and obese (O)-iPSCs are compared with publicly available RNA-seq data (GSE126848) of patients with varying degrees of NAFLD (compared in six different groups, NAFLD-BatchB/Ctrl, NAFLD-BatchA/Ctrl, NAFLD/Ctrl, NAFLD/normal, NASH/normal, NAFL/normal) and visualized as similarity percentages against each of these six groups. (**B**) Normalized log2 fold changes for selected gene list from NAFLD data and our studied groups (as described in panel A) visualized into the heatmap.


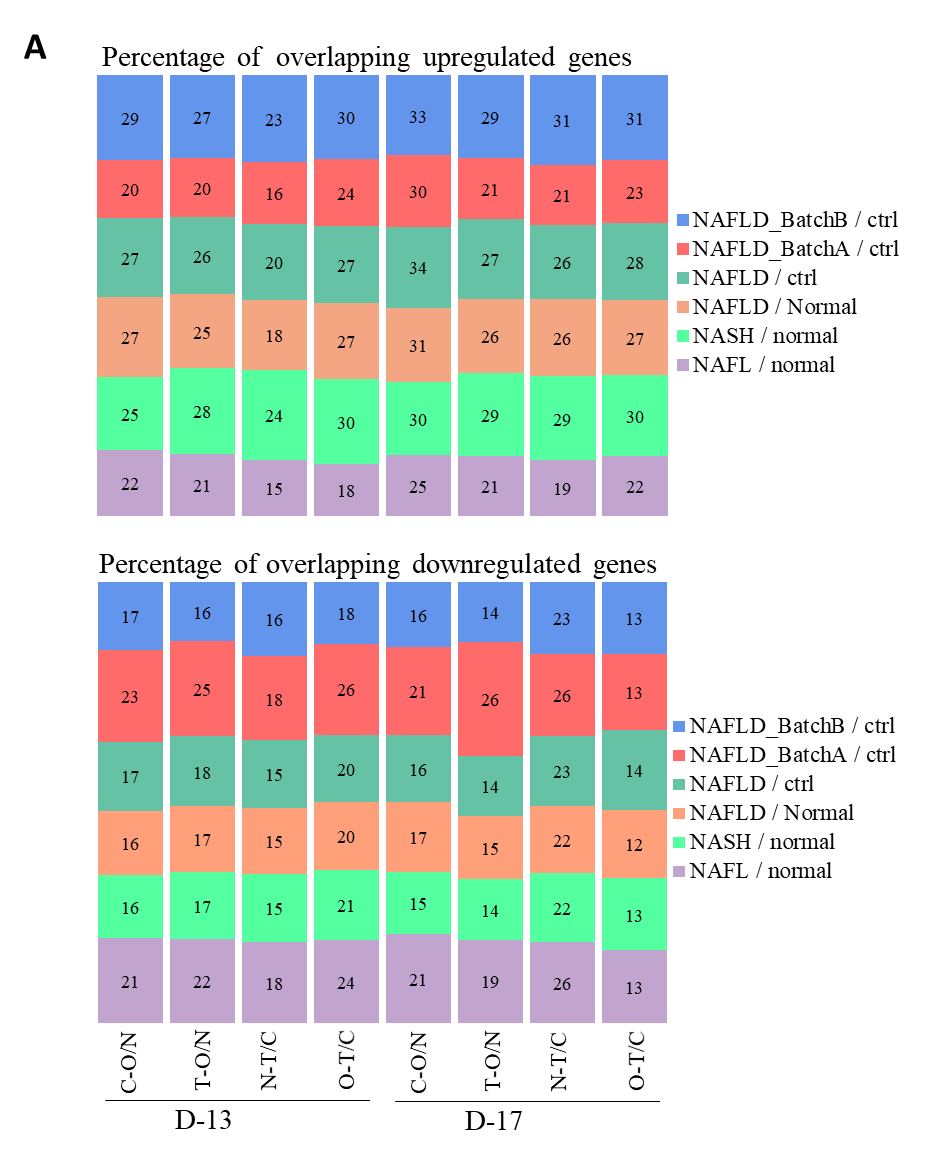


**
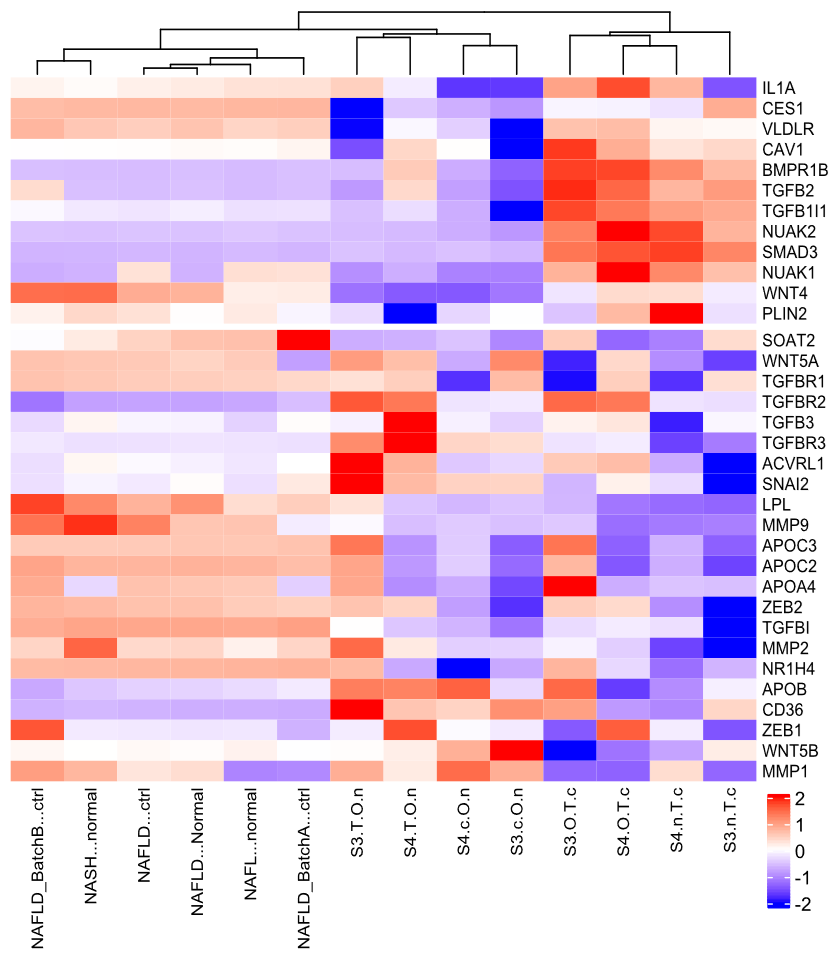
B**

**Supplementary table 1:** A list of oligonucleotide sequences used for ChIP-qPCR.

| Primer Name | Sequence |
| --- | --- |
| CAV1 –Ch- forward | 5′-GTCAACCGCGACCCTAAACA-3′ |
| CAV1 –Ch- reverse | 5′-AAGGATGCACGGGCTAACTG-3′ |
| MMP9 –Ch- forward | 5′-TCATCATGTATTGGCCCCCA-3′ |
| MMP9 –Ch- reverse | 5′-GGCCATTGGAAGTCAGGGATA-3′ |
| CD36 –Ch- forward | 5′-TGTTAAAGAGACACTGGTAAGGGT-3′ |
| CD36 –Ch- reverse | 5′-TGTGGACCAATGACCATCTGA-3′ |
| NUAK2 –Ch- forward | 5′-AGGTTTGGCAGAGGACACTC-3′ |
| NUAK2 –Ch- reverse | 5′-TGGAGTGGCTTCTATGGGTG-3′ |
| TGFBR3 –Ch- forward | 5′-GACTTCTCCCAAACCGCTCA-3′ |
| TGFBR3 –Ch- reverse | 5′-GGCGAGGGCAGAGGATAGTA-3′ |
| TBR1–Ch- forward | 5′-TCATGAGGGCAAATGGCAGT-3′ |
| TBR1-Ch- reverse | 5′-AGCTGTTGAATCGTTGGGGT-3′ |

**Supplementary table 2:** A list of antibodies used in this study.

| OCT4 (CST) | CST, Cat. #2750 |
| --- | --- |
| NANOG | CST, Cat. #4893 |
| FOXA2 (CST) | CST, Cat. #3143 |
| SOX17 (CST) | CST, Cat. #81778 |
| AFP (Abcam) | Abcam, ab3980 |
| HNF4Alpha (CST) | CST, Cat. #3113 |
| ALBUMIN (Sigma) | Sigma Aldrich, CAS No.: 9048-46-8 |
| E-Cadherin (Abcam) | Abcam, ab231303 |
| BrdU | R&D Systems, Cat. No.: MAB7225 |
| PCAF | CST, Cat. No.: 3378 |
| Histone H3 (Abcam) | Abcam, ab1791 |
| PAN-Acetyl H3 (Millipore) | Millipore, Cat. No.: 06-599 |
| H3K4Me2 | Millipore, Cat. # 07-030 |
| H3K4Me3 | Diagenode, Cat. No.: C15200152 |
| H3K9Ac | Abcam, ab177177 |
| H3K18Ac | Abcam, ab1191 |
| Total H4 | Diagenode, Cat. No.: C15410156 |
| H4K16Ac | Diagenode, Cat. No.: C15200219 |
| GAPDH | CST, Cat.# 2118 |
| Goat Anti Mouse Alexa 594 | Abcam, ab150120 |
| Goat Anti Rabbit Alexa 488 | Abcam, ab150077 |
| GAR-HRP | Jackson Immuno Research, 115-035-144 |
| GAM-HRP | Jackson Immuno Research, 115-035-166 |
